# Overview and evaluation of 15 years of Creutzfeldt-Jakob disease surveillance in Belgium, 1998-2012

**DOI:** 10.1186/s12883-015-0507-x

**Published:** 2015-12-02

**Authors:** Amber Litzroth, Patrick Cras, Bart De Vil, Sophie Quoilin

**Affiliations:** Unit of Epidemiology of Infectious Diseases, Operational Directory Public Health and Surveillance, Scientific Institute of Public Health, Juliette Wytsmanstreet 14, 1050 Brussels, Belgium; European Programme for Intervention Epidemiology Training (EPIET), European Centre for Disease Prevention and Control (ECDC), Stockholm, Sweden; Department of Neurology, Antwerp University Hospital, Wilrijkstreet 10, 2650 Edegem, Belgium; Institute Born Bunge, University of Antwerp, Antwerp, Belgium; Laboratory of Neurology, Translational Neurosciences, Faculty of Medicine and Health Sciences, University of Antwerp, Universiteitsplein 1, 2610 Wilrijk-Antwerp, Belgium

**Keywords:** Creutzfeldt-Jakob disease, Evaluation, Surveillance, Variant Creutzfeldt-Jakob disease, Acceptability, Belgium, Survey

## Abstract

**Background:**

In 1998, following the detection of variant Creutzfeldt-Jakob disease (vCJD) in the UK, Belgium installed a surveillance system for Creutzfeldt-Jakob disease (CJD). The objectives of this system were to identify vCJD cases and detect increases in CJD incidence. Diagnostic confirmation of CJD is based on autopsy after referral by neurologists. Reference centres perform autopsies and report to the surveillance system. The aim of this study was to assess whether the system met its objectives and to assess its acceptability.

**Methods:**

For 1999–2010, we linked surveillance data with hospital discharge data. We calculated the proportion of CJD suspected patients who died in hospitals and were captured by the surveillance system. We surveyed stakeholders on knowledge of the surveillance system, referral practices and acceptability. We compared proportions using the chi-square test and investigated variables associated with capture using a multivariable logistic regression model.

**Results:**

On average 60 % of hospitalised patients who died with suspected CJD were captured by the surveillance system. This proportion did not significantly differ over the years (p = 0.1). The odds of capture significantly decreased with every 1 year increase in age (OR = 0.95, 95 % CI 0.92–0.98, p = 0.001). Eleven percent of surveyed neurologists would not refer suspect vCJD cases for autopsy, nor contact a reference centre for diagnostic support. Sixty-one percent of surveyed neurologists were not familiar with the surveillance system. Awareness of the existence of the system did not impact referral behaviour (p = 0.18). CJD and vCJD surveillance were considered important by the majority of stakeholders.

**Conclusions:**

Although 40 % of the suspect CJD cases were not referred for autopsy, our data suggest that the Belgian CJD surveillance system meets one of its main objectives: it can detect changes in CJD incidence. However, we do not have sufficient evidence to conclude that the system meets its second objective of detecting vCJD cases arising in Belgium. Although not well known, the system was considered acceptable by its stakeholders.

## Background

Creutzfeldt-Jakob disease (CJD) is a transmissible spongiform encephalopathy (TSE) or prion disease that causes progressive brain degeneration. Estimated incidence worldwide ranges from 1 to 1.5 cases per million per year [1, 2]. CJD is always fatal. No therapy or vaccine is available. Four forms are known according to their aetiology: sporadic (sCJD), familial (fCJD), iatrogenic (iCJD) and variant (vCJD). Worldwide, the sporadic form accounts for 87 % of CJD cases, the familial for 8 % and the iatrogenic for less than 5 % [2]. Definite diagnosis of CJD is based on identification of the classical neuropathological triad: neuronal loss, gliosis and spongiform degeneration, and on identification of prion protein deposition in the brain. Therefore definitive diagnosis requires post-mortem neuropathological examination. Differentiation of vCJD and the other CJD forms also requires autopsy.

In 1996, vCJD was first detected in the UK [3]. This form of CJD was soon linked to bovine spongiform encephalopathy (BSE), a related disease that had been causing an epidemic in cattle in the UK since 1986. Evidence suggested that human vCJD cases were infected through consumption of contaminated animal products [4]. Variant CJD cases differ from other CJD cases. The patient’s median age at death is 28 years [5], compared with 62–68 years for sporadic CJD [6–8]. In addition, the median disease duration among vCJD cases is 14 months [5], compared with 4–6 months for other CJD’s [6–8]. Between 1995 and 2013, 228 people worldwide died from vCJD, of whom 177 had lived in the UK [9]. The peak of the epidemic was reached in 2000, and the annual incidence has been decreasing since [9, 10]. Although the appearance of a smaller second peak in the future cannot be excluded [11], the public health importance of a vCJD pandemic has decreased in the 2000s.

In 1998, Belgium initiated a surveillance system for all forms of CJD. Initially, the objective of this surveillance was to detect vCJD cases in order to identify risk factors, trace back the source of infection, identify possible cases sharing the same exposure and prevent secondary transmission through blood products and transplants. The second objective was to detect increases in the CJD incidence, which could be an early warning signal for the emergence of new prion diseases or for new modes of transmission [12, 13]. Up to December 2014, no vCJD case has been diagnosed in Belgium and the decrease in public health relevance may have led to a decrease in the proportion of clinically suspected CJD cases referred for autopsy [7].

We evaluated if the Belgian CJD surveillance system is capable of reaching its objectives to detect vCJD cases and significant increases in the CJD incidence. We also assessed the system’s data quality, acceptability and simplicity.

## Methods

### Case definitions

The 1998 Rotterdam criteria, with a 2010 modification, classify sporadic, familial and iatrogenic CJD [14–16] (Table 1). A possible case requires clinical evidence. Probable cases require additional electroencephalogram (EEG), magnetic resonance imaging (MRI) or laboratory suggestive evidence. Confirmed cases require neuropathological confirmation.

**Table 1:** Diagnostic criteria CJD based on the Rotterdam criteria and the UK criteria

SPORADIC CDJ		
Criteria		
I		Rapidly progressive dementia
II	A	Myoclonus
	B	Visual or cerebellar problems
	C	Pyramidal or extrapyramidal features
	D	Akinetic mutism
III		CJD-typical periodic sharpwave complexes in electroencephalography
IV^a^		High signal in caudate/putamen on MRI brain scan
Classification		
Confirmed sporadic CJD		Neuropathological/immunocytochemical confirmation
Probable sporadic CJD		I *and* 2 of II *and* III OR I *and* 2 of II *and* IV^a^ OR possible sporadic CJD *and* positive 14-3-3
Possible sporadic CJD		I *and* 2 of II *and* duration < 2 years
^a^As of 2010		
IATROGENIC CJD		
Progressive cerebellar syndrome in a pituitary hormone recipient *OR* sporadic CJD with a recognised exposure risk, e.g., dura mater transplant		
FAMILIAL CJD		
Confirmed or probable CJD plus confirmed or probable CJD in a first-degree relative *O*R neuropsychiatric disorder plus disease-specific PRNP mutation		
VARIANT CDJ		
Criteria		
I	A	Progressive neuropsychiatric disorder
	B	Duration of illness > 6 months
	C	Routine investigations do not suggest an alternative diagnosis
	D	No history of potential iatrogenic exposure
	E	No evidence of a familial form of TSE
II	A	Early psychiatric features^a^
	B	Persistent painful sensory symptoms^b^
	C	Ataxia
	D	Myoclonus or chorea or dystonia
	E	Dementia
III	A	EEG does not show the typical appearance of sporadic CJD in the early stages of illness^c^
	B	Bilateral pulvinar high signal on MRI scan
IV	A	Positive tonsil biopsy
Classification		
Confirmed variant CJD		IA and neuropathological confirmation of vCJD^d^
Probable variant CJD		I and 4/5 of II and IIIA and IIIB OR 1 and IVA
Possible variant CJD		I and 4/5 of II and IIIA

^a^Depression, anxiety, apathy, withdrawal, delusions

^b^Including both frank pain and/or dysaesthesia

^c^Generalised triphasic periodic complexes at approximately one per second

^d^Spongiform change and extensive PrP deposition with florid plaques, throughout the cerebrum and cerebellum

The 2000 UK criteria classify vCJD cases [17] (Table 1). Possible vCJD cases rely on clinical evidence. Probable vCJD cases require an additional characteristic brain magnetic resonance imaging (MRI) or positive tonsil biopsy. Confirmed vCJD cases require neuropathological confirmation.

For the purpose of this evaluation, we defined suspect CJD cases as patients that died with the suspicion of CJD, regardless of case classification criteria.

### Organization of the surveillance system

The Belgian CJD surveillance system covers probable and confirmed cases of CJD in people with the Belgian nationality. Seven academic centres of neurology/neuropathology are appointed as CJD reference centres. The Scientific Institute of Public Health (WIV-ISP) acts as the coordinator and maintains the surveillance database [8]. Neurologists that suspect CJD in a patient can refer the patient to a reference centre, which reports probable cases. Four of these centres can confirm cases by autopsy. They report on all autopsies of suspect CJD cases, regardless of whether the CJD diagnosis was confirmed or not. The surveillance system reimburses these autopsies. In cases where the patient or his family refuses referral to a reference centre, the neurologist can report the probable case. Reference centres and neurologists report cases and autopsies using a 4-page paper form. The form collects information on personal and demographic characteristics, treating physicians, signs, symptoms, results of diagnostic procedures (including stored specimens) and risk factors. The completed form is sent by regular mail to the WIV-ISP, which is responsible for collecting, storing and processing the data. Data are analysed yearly and a report is published on the WIV-ISP website.

### Analysis and completeness of surveillance data

For 1998–2012, we analysed the Belgian surveillance data. We calculated the annual number of reported autopsies, the reported probable and confirmed CJD cases, the yearly incidence of probable and confirmed CJD cases and the age-standardized yearly incidence of probable and confirmed CJD cases according to the age distribution in the World Health Organization (WHO) World standard population [18]. We described the final diagnosis of patients that were found negative for CJD in the autopsy.

Data completeness was assessed for all reported autopsies and probable cases for the years 1998–2012 for the following variables: postcode, sex, date of birth, date of death, disease duration, symptoms at onset, EEG result, 14-3-3 protein test result, MRI result, prion protein (PrP) gene analysis result and familial history of CJD. Data completeness was defined as the proportion of data that is not stated as ‘missing’ or ‘unknown’. We calculated the annual completeness per variable, the annual completeness over all variables and the total completeness per variable over the period 1998–2012. For the years 1998–2012, we looked for trends in annual completeness per variable and in annual completeness over all variables.

### Estimation of suspect cases captured by the surveillance system

For 1999–2010, Belgian surveillance data were linked with hospital discharge data. Records were linked based on age, gender and postal code. Records with more than one possible link were excluded. We calculated the annual proportion of suspect cases that died in hospital with CJD as primary or secondary diagnosis (ICD 9 code: 046.1) for which autopsy was performed in a reference centre. We assessed differences in the proportion of autopsies performed between the year of death, the province, gender and those with CJD as primary or as secondary diagnosis. The effect of age of the patient on the performance of an autopsy was assessed. For 1999–2010, we looked for a trend in the proportion of suspect cases that died in hospitals and underwent autopsy.

These procedures were repeated for suspect cases that died in hospital with CJD as primary or secondary diagnosis and were captured by the surveillance system either through an autopsy either as a probable case.

### Online surveys

We developed three online surveys: for Belgian neurologists, for persons responsible for the reference centres and for public health officials.

Neurologists were invited through two professional organizations. They were asked about their knowledge of the surveillance system, their referral behaviour and their perception of the importance of vCJD and CJD surveillance. They were also asked about changes in participation or interest in CJD surveillance since 1998, on their reporting preferences and on limitations of the system.

We sent invitations by email to the persons responsible for the reference centres. They were asked about their perception of the importance of vCJD and CJD surveillance, on their reporting preferences and on limitations of the system.

We contacted public health officials through their governmental organisations and asked them to further distribute the email. They were asked about their perception of the importance of vCJD and CJD surveillance.

### Ethics committee statement

The Ethics Committee of the Antwerp University Hospital reviewed the study and granted a waiver of informed consent, as the date are collected within the context of health surveillance as requested by European legislation.

### Statistical analysis

Data were analysed using Microsoft Excel and Stata 12 (StataCorp). We compared proportions using the chi-square test and checked for trends using the Cuzick non-parametric test for trends. The effect of a 1 year increase in age on the performance of an autopsy and on total capture by the surveillance system was assessed by calculating odds ratios (OR) and 95 % confidence intervals (CI) in a logistic regression model. Variables associated with autopsy with a *p*-value <0.2 in the univariate analysis were included in a backward stepwise multivariable logistic regression model. A similar model was built for variables associated with total capture by the surveillance system. A *p*-value <0.05 was considered significant.

## Results

### Belgian CJD surveillance 1998–2012

**Fig. 1:**
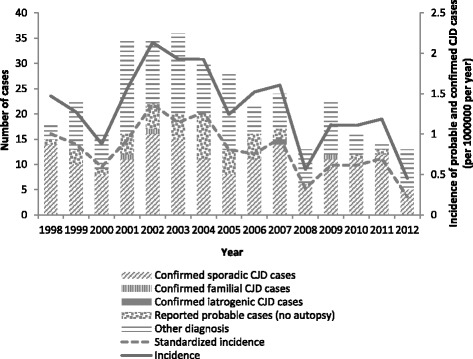
CJD incidence and autopsy results of suspected CJD cases by case classification, 1998–2012, Belgium

In 1998–2012, 304 autopsies of Belgian suspect CJD cases were reported to the surveillance system. Of all autopsies, 167 (55 %) resulted in CJD diagnosis. Of these, 163 (98 %) were sCJD cases, three (1.4 %) fCJD and one (0.6 %) iCJD. In addition, 42 probable CJD cases, among which two probable fCJD cases, were also reported to the system (Fig. 1). Recorded incidence ranged from 0.5 to 2.1 cases per million inhabitants per year (average 1.3), and age-standardized incidence ranged from 0.2 to 1.4 cases per million inhabitants per year (average 0.8) (Fig. 1).

Among the 137 (45 %) autopsies that were found negative for CJD, 31 (23 %) were cases of Alzheimer’s dementia, 18 (13 %) of dementia (not further specified), 16 (12 %) of Lewy body dementia, 8 (6 %) of encephalitis, 24 (18 %) had no final diagnoses and 40 (29 %) had various other diagnoses.

### Data quality

We calculated data completeness for 346 reports of autopsies and probable cases. For 1998–2012, the annual completeness over all variables ranged from 53 to 89 % (data not shown). The variables with lowest total completeness over this period were MRI result (49 %) and PrP gene analysis result (33 %). The variables with highest total completeness over this period were sex (100 %), date of birth (97 %), date of death (96 %) and postal code (91 %). Total completeness over this period of the other variables ranged between 60 and 80 %. For 1998–2012, a significant downward trend in annual completeness was observed in the following variables: symptoms at onset (p = 0.004), EEG result (p = 0.023) and familial history of CJD (p = 0.004). For 1998–2012, a significant upward trend in annual completeness was observed in the following variables: date of birth (p = 0.034) and date of death (p = 0.028). For the other variables we observed no trend in annual completeness over the period 1998–2012. The annual completeness over all variables decreased significantly from 1998 to 2012 (p = 0.024).

### Estimation of suspect cases captured by the surveillance system

In 1999–2010, the hospital discharge database contained 241 suspect cases. Of these, 122 (51 %) underwent an autopsy in a reference centre. The annual proportion ranged from 35 % in 2010 to 65 % in 2002 (Table 2), with no trend (p = 0.14). In univariate analysis we did not detect a significant difference in proportion referred for autopsy between years (p = 0.65), province (p = 0.11), gender (p = 0.74) and those with CJD as primary or as secondary diagnosis (p = 0.22). The odds of undergoing an autopsy significantly decreased with every 1 year increase in age (OR 0.97, 95 % CI 0.94–0.99, p = 0.01). Only the effect of age was significant in the multivariable analysis (OR 0.97, 95 % CI 0.94–0.99, p = 0.01).

**Table 2:** Annual suspect CJD cases by way of capture by the Belgian CJD surveillance system, 1999-2010

Year	Number of suspect CJD cases in hospital discharge data	Number of autopsies performed on suspect CJD cases (% of total suspect cases)	Number of suspect CJD cases captured by surveillance system (autopsy or probable) (% of total suspect cases)	Number of suspect CJD cases not captured by the surveillance system (% of total suspect cases)
1999	15	9 (60)	10 (67)	5 (33)
2000	14	7 (50)	7 (50)	7 (50)
2001	19	10 (53)	14 (74)	5 (26)
2002	20	13 (65)	16 (80)	4 (20)
2003	24	14 (58)	17 (71)	7 (29)
2004	24	10 (42)	15 (63)	9 (37)
2005	17	8 (47)	10 (59)	7 (41)
2006	20	9 (45)	13 (65)	7 (35)
2007	25	15 (60)	15 (60)	10 (40)
2008	15	6 (40)	6 (40)	9 (60)
2009	17	10 (59)	10 (59)	7 (41)
2010	31	11 (35)	11 (35)	20 (65)
Total	241	122 (51)	144 (60)	97 (40)

Out of 241 patients that died in hospital with CJD as a primary or secondary diagnosis, 144 (60 %) were captured by the surveillance system (autopsy or probable case). The annual proportion varied between 35 % in 2010 and 80 % in 2002 (Table 2). This proportion showed a downward trend (p = 0.004). In univariate analysis we did not detect a significant difference in this proportion between years (p = 0.1), gender (p = 0.66) and between groups with CJD as primary or as secondary diagnosis (p = 0.15). We detected a significant difference between the provinces (p = 0.023) and the odds of capture significantly decreased with every 1 year increase in age (OR 0.95, 95 % CI 0.92–0.98, p = 0.001). Only the effect of age remained significant in the multivariable analysis (OR 0.95, 95 % CI 0.92–0.98, p = 0.001).

### Survey response

We could not calculate response proportions for neurologists, because we did not have access to the email distribution lists of the organisation of neurologists. As a proxy figure for the neurologists, we used the number of registered neurologists available from the national institute for health insurance.

Of 533 neurologists registered in Belgium, 72 (14 %) completed the survey. Of seven contacted reference centres, four (57 %) submitted a completed survey. Thirteen public health officials completed the survey, since we asked to forward the email within their organisations, no response proportion could be calculated.

### Survey results on referral behaviour of neurologists

Among the 72 neurologists who completed the survey, 28 (39 %) were aware of the existence of the surveillance system. However, 61 (85 %) neurologists would refer all suspect CJD cases or contact a reference centre for diagnostic support. Three neurologists (4 %) would only refer vCJD cases. Among those who would not refer cases nor contact a reference centre, three (38 %) were unaware of the existence of the centres, and five (63 %) did not see the added value of referral (Table 3). Being aware of the existence of the surveillance system did not impact the referral behaviour of the neurologists (p = 0.18).

**Table 3:** Knowledge of the Belgian CJD surveillance system and referral practices reported by Belgian neurologists

Topic of the question	Neurologists’ response	Number	Percent
Knowledge of the existence of the surveillance system (N = 72)	Yes	28	39
Referral behaviour in case of suspected CJD (N = 72)	Do not refer or contact for support	8	11
	Would refer if vCJD is suspected	3	4
	Only contact for support	11	15
	Always refer for autopsy	15	21
	Always refer immediately	4	6
	Always contact for support and refer for autopsy	31	43
Reasons no referral/no contact for support (N = 8)	Unaware of the existence of these centres	3	38
	Do not see the added value	5	63

Legend: Knowledge of the Belgian CJD surveillance system and referral practices as reported by Belgian neurologists (N = 72) in an online survey, 2013, Belgium

### Survey results on acceptability and simplicity of the surveillance system

**Fig. 2:**
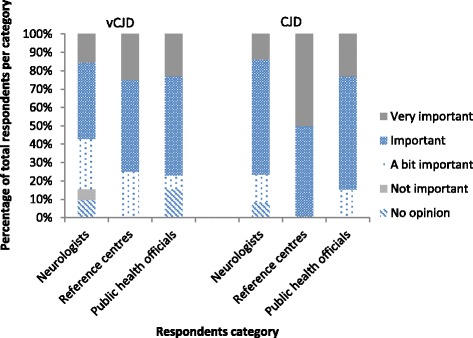
Importance of vCJD and CJD surveillance as reported by stakeholders, 2013, Belgium

The majority of stakeholders surveyed, including 41 (57 %) neurologists, three (75 %) responsibles of reference centres and 10 (77 %) public health officials, considered variant CJD surveillance important. The majority of stakeholders surveyed, including 55 (76 %) neurologists, four (100 %) responsibles of reference centres and 11 (85 %) public health officials, considered CJD surveillance important (Fig. 2). Nine (13 %) neurologists reported a decrease in their participation or interest in CJD surveillance.

Of 72 neurologists and reference centres, six (8 %) had no opinion regarding reporting procedures, five (7 %) preferred the currently used paper form and 65 (86 %) preferred electronic reporting. Of the latter, 39 (51 %) preferred an online questionnaire.

Among the 72 neurologists, 16 (22 %) emphasized the need for better visibility of the system and regular feedback to the data providers.

## Discussion

This evaluation indicated that in 1999–2010, a stable proportion of suspect CJD cases who died in hospitals underwent an autopsy in a reference centre or was captured by the surveillance system as either an autopsy or a probable case. The proportion of suspect CJD cases captured by the surveillance system showed a downward trend. The older the suspected case was, the less likely he was to have an autopsy performed or to be captured by the surveillance system. Among neurologists who completed the survey, 89 % would refer a vCJD case to a reference centre or contact a reference centre for diagnostic support. In 1998–2012, data quality decreased. The majority of stakeholders surveyed considered CJD and vCJD surveillance important and preferred online reporting. The main reported limitation to the system was its lack of visibility and feedback to the neurologists. Only 39 % of surveyed neurologists were aware of the system’s existence.

A stable referral of suspect cases for autopsy is key for detection of increases in CJD incidence that could be the sign of an undetected event. Increases in the measured incidence of sCJD over time have indeed previously been attributed to enhanced surveillance [1, 8, 19, 20] and studies have shown that the proportion of suspect CJD cases that is referred for autopsy, predicts the measured incidence of CJD [21, 22]. Changes in the measured incidence can therefore be caused by a non-stable yearly proportion of suspect cases referred for autopsy or by a true change in the incidence. Our data show that in Belgium for 1999–2010, the proportion of suspect cases referred for autopsy was similar over the years, around 50 %. The proportion of suspect cases captured by the surveillance system as an autopsy or a probable case was also similar over the years around 60 %. These findings suggest that increases in the measured CJD incidence would very likely reflect true increases in CJD incidence. Moreover, the stability between the Belgian provinces of the proportion of suspect cases referred for autopsy, decreases the chance of misidentifying geographical clusters due to locally increased surveillance as previously reported in other countries [6, 21, 22]. Our data did show a significant downward trend in the proportion of suspect cases captured by the surveillance system. Although this trend has not yet resulted in significant differences over the years, a continuation of this trend will inevitably do so, so it is important to monitor referral in the future. The reason for the decrease in capture by the surveillance system with increasing age is not clear. Families may be more reluctant in accepting an autopsy for an elderly person or doctors may be less focussed on having a final diagnosis in these cases. Although identifying the reason for this falls outside the scope of our study, this decrease may result in an underestimation of CJD in the older people and should be investigated further. Thus, the surveillance system meets one of its main objectives as it is likely to detect true increases in CJD incidence in Belgium, but monitoring the stability of the referral, both for autopsy as for the reporting of probable cases, remains essential.

Although the public health relevance of a vCJD epidemic has decreased in recent years and the surveillance system is not well known, our investigation of neurologists’ referral behaviour suggests that they were still aware of the importance of detecting vCJD cases and contacting a reference centre. Among those neurologist that responded to the survey, the vast majority (89 %) would refer suspect vCJD cases to a reference centre or contact a reference centre. Moreover, the younger age of vCJD patients and the longer disease duration would render detection of vCJD cases by the surveillance system more likely, even if the neurologist does not contact the reference centre immediately. However, based on this evaluation, we cannot exclude the possibility that a vCJD case arising in Belgium remains undetected. Therefore, we do not have sufficient evidence to conclude that the surveillance system meets its second main objective of detecting vCJD cases arising in Belgium.

Only one third of neurologists were aware of the existence of the surveillance system and 22 % believed the system lacks visibility and regular feedback to the data providers. However, this does not indicate that neurologists do not refer cases to a reference centre. The lack of association between referral behaviour of neurologists and awareness of the surveillance system suggests that referring cases or contacting a reference centre for diagnostic support is not done to contribute to the surveillance system, but as part of good clinical practices. Although the system is not well known, the majority of data providers and public health officials considered both vCJD and CJD surveillance at least important. This suggests that CJD and vCJD surveillance are acceptable, even in absence of the threat of a large vCJD epidemic. Only nine (13 %) neurologists reported a decrease in their participation or interest in CJD surveillance. Our results suggest this may have impacted data quality, which has been dropping since 1998.

An important limitation of this evaluation lies in the unknown representativeness of survey respondents. Those with a special interest in CJD may have been more likely to respond. The impact of this on our evaluation cannot be estimated, and therefore the results of the survey should be interpreted with caution and verified where possible. An example of the responder bias might be represented in the difference between measured referral of suspect cases and self-reported referral by neurologists. Sixty-eight percent of respondents said they referred suspect CJD cases for autopsy. However, when linking the hospital discharge data with the cases referred for autopsy, only 50 % of cases were referred. However, a possible alternative explanation for the difference between the estimated referral and the self-reported referral may lie in a reporting error on hospital level or an autopsy refusal of the family. Checking death records of suspect CJD cases that did not undergo autopsy in a reference centre would provide us with more insight into referral practices and would perhaps allow us to estimate the Belgian incidence more precisely. Moreover, it could clarify the decrease in capture by the surveillance system with increasing age.

Another limitation lies in the fact that some cases that were referred for autopsy did not come from a hospital setting. Indeed, not all patients that undergo autopsy have died in hospital as some of them die at home or in nursing homes. In this evaluation, we were not able to estimate the proportion of suspect CJD cases referred for autopsy from these settings. Therefore, we cannot be certain that referral behaviour in another setting has not changed over the years. However, we consider it unlikely that referral behaviour would remain stable in one setting and not in another.

## Conclusions

In conclusion, the Belgian CJD surveillance system meets one of its two main objectives: the system is capable of detecting changes in CJD incidence in time, both at national and regional level. Although self-reported referral behaviour of the Belgian neurologists indicates that vCJD cases are likely to be captured by the surveillance system, this evaluation does not give the necessary evidence that the system meets its second objective of capturing vCJD cases. The system was acceptable to data providers and public health officials. Nevertheless, data providers consider the lack of regular feedback and the current way of reporting as the two main limitations to the system. Moreover, we found that half of the suspect CJD cases were not referred for autopsy and we found a decreasing trend in total capture over the years and a decreasing capture with age. Therefore, we recommend to (a) revise the data reporting through implementation of electronic tools (b) provide regular feedback to reporting neurologists to enhance their contribution, and (c) explore the reasons for not undergoing autopsy, for example by performing a validation study on the CJD suspected deaths in hospital that do not undergo autopsy.

## Abbreviations

CJD: Creutzfeldt-Jakob disease
EEG: electroencephalogram
fCJD: familial Creutzfeldt-Jakob disease
iCJD: iatrogenic Creutzfeldt-Jakob disease
MRI: magnetic resonance imaging
sCJD: sporadic Creutzfeldt-Jakob disease
TSE: transmissible spongiform encephalopathy
vCJD: variant Creutzfeldt-Jakob disease
WIV-ISP: Belgian Scientific Institute of Public Health
